# Building Emotional Awareness and Mental Health (BEAM): study protocol for a hybrid implementation-effectiveness trial of the BEAM app-based program for parents with clinical mental health problems

**DOI:** 10.1186/s12888-025-06964-4

**Published:** 2025-06-02

**Authors:** Kaeley M. Simpson, Robert J. W. McHardy, J. Grace Zhou, Sydney Levasseur-Puhach, Cynthia M. Côté, Millie Braun, Fiona Clement, Anna L. MacKinnon, Nathan Nickel, Tracie O. Afifi, Gerald F. Giesbrecht, Ryan Giuliano, Laurence Y. Katz, Lauren E. Kelly, Terry P. Klassen, Catherine Lebel, Aislin Mushquash, Kristin Reynolds, Elizabeth Decaire, Wajihah Mughal, Frances Chartrand, Olena Kloss, Jennifer M. Hensel, James M. Bolton, Jo Ann M. Unger, Mandy Archibald, Philip Fisher, Ashley Pharazyn, Lianne Tomfohr-Madsen, Leslie E. Roos

**Affiliations:** 1 University of Manitoba, Winnipeg, Canada; 2 Independent Affiliation, Winnipeg, Canada; 3 University of Calgary, Calgary, Canada; 4 University of Montreal, Montreal, Canada; 5 Children & Hospital Research Institute of Manitoba, Winnipeg, Canada; 6 Lakehead University, Thunder Bay, Canada; 7 First Nations Health and Social Secretariat of Manitoba, Winnipeg, Canada; 8 Manitoba Métis Federation, Winnipeg, Canada; 9 9 Stanford University, Stanford, USA; 10 University of British Columbia, Vancouver, Canada

**Keywords:** Parenting, Mental health, Depression, Anxiety, Psychoeducation, App-based intervention

## Abstract

**Background:**

Children are highly sensitive to adversity during their first five years of life, with exposure to chronic parental mental illness (MI) consistently linked to socio-emotional impairments and mental health problems in children. Children born during the COVID-19 pandemic were exposed to unprecedented levels of parental distress, with parental MI reported at three times the pre-pandemic rates. This situation underscored a pressing need for scalable solutions to foster positive mental health and developmental outcomes for a generation of children. In response, we developed the Building Emotional Awareness and Mental Health (BEAM) program, an innovative mobile health (mHealth) solution for parents of young children. Clinical trials to date evaluating BEAM have shown promising results, demonstrating reductions in parent depression, suicidality, anxiety, and harsh parenting practices. This trial involves an effectiveness-implementation hybrid design with co-primary aims of (1) determining BEAM’s effectiveness in improving parent mental health, and (2) evaluating the implementation of BEAM in the community through metrics such as feasibility, acceptability, and uptake. This trial’s secondary aim is to measure BEAM’s effectiveness in improving short-term child mental health and developmental outcomes using primary data and long-term psychosocial family outcomes using administrative data. A final exploratory aim of this trial will measure the cost-utility of delivering BEAM relative to extant health programming.

**Methods:**

A single arm trial with repeated measures will be used to evaluate the effectiveness of implementing the BEAM intervention in the community with a sample of 400 parent participants with a child aged 24–71 months. Participants must self-report moderate to severe symptoms of depression, anxiety, parenting stress, and/or anger at time of enrolment (T0) and live in the province of Manitoba, Canada. Individuals will be recruited through four streams including the (1) Manitoba Crisis Response Services, (2) primary care offices (paediatricians and/or general practitioners), (3) Manitoba family community organizations and child care centres, and (4) social media. Study participants will complete 12 weeks of psychoeducation modules, with access to an online social support forum and check ins with a peer coach. Assessments of parent and child mental health symptoms will occur at pre-test before BEAM begins (T1), immediately after the last week of the BEAM intervention (post-test, T2), 6-month follow-up (T3), and 12-month follow-up (T4).

**Discussion:**

The BEAM program offers a promising solution to address elevated parental mental health symptoms, parenting stress, and related child functioning concerns. The present implementation trial aims to extend the groundwork established by an open pilot trial and RCT of the BEAM program, in a next step of testing BEAM’s readiness for nationwide scaling.

**Trial registration:**

ClinicalTrials.gov NCT06455397. Registered on June 11, 2024.

## Administrative information

Note: the numbers in curly brackets in this protocol refer to SPIRIT checklist item numbers. The order of the items has been modified to group similar items (see http://www.equator-network.org/reporting-guidelines/spirit-2013-statement-defining-standard-protocol-items-for-clinical-trials/).

**Table:** 

Title	Building Emotional Awareness and Mental Health (BEAM): Study protocol for a hybrid implementation-effectiveness trial of the BEAM app-based program for parents with clinical mental health problems
Trial registration {2a and 2b}.	The trial was registered with Clinicaltrials.gov on June 11, 2024. All items from the WHO Trial Registration Dataset can be found within the protocol.
Protocol version {3}	[June 19, 2024] (Version 1)
Funding	This work was supported by funding from the Canadian Institutes of Health Research (#185,112), the Children’s Hospital Foundation of Manitoba & Sobeys Foundation (Family of Support Initiative).
Author details {5a}	Kaeley M. Simpson, University of Manitoba Robert J. W. McHardy, University of Manitoba J. Grace Zhou, University of Manitoba Sydney Levasseur-Puhach, University of Manitoba Cynthia C. Côté, University of Manitoba Millie Braun, Independent Affiliation Fiona Clement, University of Calgary Anna L. MacKinnon, University of Montreal Nathan Nickel, University of Manitoba Tracie O. Afifi, University of Manitoba Gerald F. Giesbrecht, University of Calgary Ryan Giuliano, University of Manitoba Laurence Y. Katz, University of Manitoba Lauren E. Kelly, University of Manitoba Terry P. Klassen, Children’s Hospital Research Institute of Manitoba Catherine Lebel, University of Calgary Aislin Mushquash, Lakehead University Kristin Reynolds, University of Manitoba Elizabeth Decaire, First Nations Health and Social Secretariat of Manitoba Wajihah Mughal, Manitoba Métis Federation Frances Chartrand, Manitoba Métis Federation Olena Kloss, Manitoba Métis Federation Jennifer M. Hensel, University of Manitoba James M. Bolton, University of Manitoba Jo Ann M. Unger, University of Manitoba Mandy Archibald, University of Manitoba Philip Fisher, Stanford University Ashley Pharazyn, University of Manitoba Lianne Tomfohr-Madsen, University of British Columbia* Leslie E. Roos, University of Manitoba* *co-last authors
Name and contact information for the trial sponsor {5b}	Leslie Roos Department of Psychology, University of Manitoba University of Manitoba, 66 Chancellors Cir, Winnipeg, MB, R3 T 2 N2. E-mail: leslie.roos@umanitoba.ca
Role of sponsor {5c}	This funding source had no role in the design of this study and will not have any role during its execution, analyses, interpretation of the data, or decision to submit results.

## Introduction

### Background and rationale {6a}

The initial five years of a child’s life mark a critical developmental phase and a period of high sensitivity to environmental stressors, including the impact of parental mental illness (MI) and parenting stress. Research has consistently linked parental MI with a broad range of child-related issues, including irritability, sleep disturbances, and socio-emotional developmental impairments [1–4]. These adverse outcomes are often attributed to environmental factors, including parental modeling of maladaptive emotion coping strategies such as avoidance, aggression, and harsh parenting practices characterized by reactive discipline and conflictual interactions [5–7]. Notably, when parental MI is accompanied by additional stressors such as domestic conflict or financial strains, the long-term risks for children are exacerbated [8]. Further, the chronicity of parental MI has critical implications for children. When stressors and parental MI are persistent, the risk of adverse developmental outcomes for children increases, putting children at heightened risk for stress and development of their own psychopathology [9, 10]. This highlights the critical need for interventions that address parental MI and the broader spectrum of parenting stress and its multifaceted impacts on children.

Despite the need for parents to improve their stress and mental health symptoms, the majority of families do not have access to evidence-based treatments [2, 3, 11]. Previous research has documented many barriers preventing parents from accessing care. These barriers include service backlogs, long waitlists, high costs of individual therapy, lack of information of where to access interventions, perceived need for care, stigma, and overwhelming childcare demands [2, 3, 11–15]. Additionally, although evidence-based treatments exist, most interventions do not comprehensively address the mental health of both parents and children. This gap in services is significant, given meta-analytic evidence indicating that dual-generation programs, which simultaneously target parent MI and child well-being, yield impacts that are 50% larger in promoting positive child outcomes compared to programs focused solely on addressing parental MI [10]. There is a clear need to provide accessible and scalable solutions that promote positive mental health and developmental outcomes in at-risk children. mHealth (i.e., the use of mobile devices, such as cellphones and tablets, for healthcare delivery) interventions offer a potential avenue for addressing family needs and barriers to care that are an accessible and low-cost option, and research shows great promise for treating adult depression using these methods [16]. Additional emerging research highlights the efficacy of app-based programs in improving parental MI and parent–child interactions [17, 18]. However, very limited existing app-based or mHealth programs address both parental mental health and parenting skills, which indirectly targets child well-being.

### Building emotional awareness and mental health program

In response to this need, we conducted qualitative research (i.e., focus groups and individual interviews with parents with lived experience) and consulted our parent advisory board with the aim of co-developing a program that simultaneously addressed parental MI and parenting. We found that parents wanted accessible, online services grounded in expert research [19–21]. Alongside patient-partners and community providers, we then developed the BEAM (Building Emotional Awareness and Mental Health) app-based program. The BEAM program is aligned with best practices in mHealth programs including patient-driven priorities, rapid-cycle iterations to facilitate continual improvements, and a commitment to evidence-based care [22–24]. Key elements of the original BEAM program include: (1) expert-led educational videos using transdiagnostic therapy [25, 26] and emotion-focused parenting strategies [20]; (2) brief group sessions to consolidate therapeutic content and build social support [27, 28]; (3) a community forum to enhance social connection; and (4) symptom monitoring to track progress [29]. In case of a mental health or parenting-related crisis, clinical coaches also consult with the registered psychologists on the research team via phone. BEAM builds on evidence from our knowledge synthesis work that mHealth therapeutics can address parent MI and appeals to parents [22, 23, 30].

The BEAM intervention has consistently demonstrated promising outcomes across various trials to date. We first conducted an open pilot and pilot RCT which demonstrated BEAM’s efficacy in reducing MI symptoms such as depression, anxiety, anger, sleep issues, and substance use [31, 32]. In our latest phase II RCT with mothers of toddlers, the BEAM program outperformed a services-as-usual (SAU) control condition [33]. Significant improvements in parental MI symptoms including anxiety, anger, and alcohol use were observed. Additionally, BEAM was effective in reducing harsh parenting practices and negative parent–child interactions, with substantial improvements observed for families living in poverty. This trial also showed noteworthy participant engagement with retention rates (84%), comparable to in-person therapy sessions [34]. Qualitative feedback from the initial trials emphasized the positive impact of the BEAM program on mental health and parenting, leading to enhanced quality of life and improved family relationships. Participants also highlighted the value of the social support gained through the online community [31, 32].

### The present study

To further address family mental health needs, we aim to test the readiness of the BEAM program for scalability. The current study involves a hybrid effectiveness-implementation trial design to build on our previous work. Specifically, effectiveness and implementation are co-primary outcomes. We will evaluate effectiveness by examining short-term change in parent mental health symptoms using primary data and longer-term change in family health and psychosocial outcomes with linked administrative data. We will evaluate implementation using objective and subjective metrics of feasibility, acceptability, and uptake at post-intervention. Our hybrid design follows the “type 2” model [35], in which effectiveness and implementation are co-primary aims and can be tracked simultaneously as the trial progresses. This approach is consistent with our rapid-cycle program development in which we have sought to test and adapt BEAM in response to patient and provider feedback through each iteration.

**Fig. 1:**
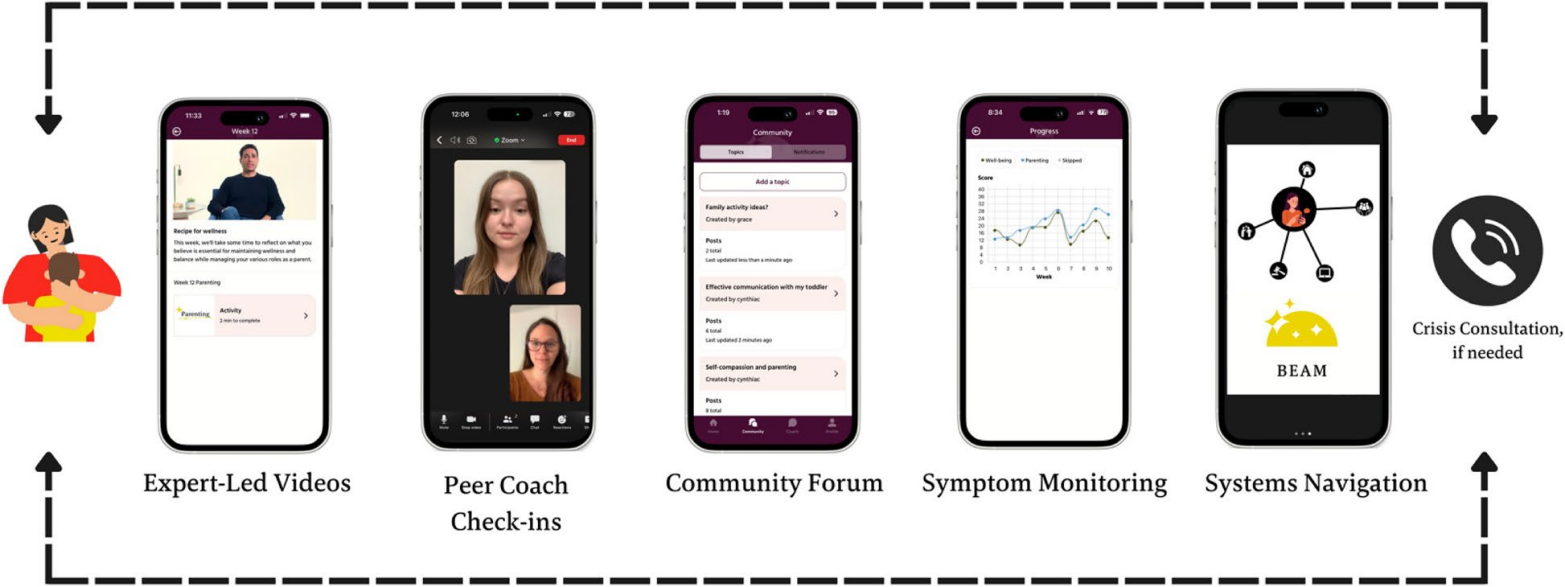
BEAM Version 2.0. Program Components. Note. Expert-led videos provide weekly psychoeducational mental health and parenting content within the BEAM app. Guided content review and individualized support is led by trained peer coaches. Symptom monitoring allows participants to complete brief weekly symptom tracking surveys in app. The community forum facilitates communication and connection with other program participants. The Systems Navigator can support participants in accessing community resources (e.g., childcare programs, legal aid, housing). Consistent with a stepped care approach, responsive crisis consultation will be provided by the BEAM Clinical Team

Through this implementation trial we aim to maximize BEAM’s accessibility, equitability, and effectiveness for future nation-wide implementation. For the current trial, we conducted an app rebuild to create BEAM Version 2.0 based on participant and parent advisory board feedback. BEAM 2.0 updates include improvements to psychoeducational video content (e.g., high-quality video production, animations, closed captioning), the mobile application user experience (e.g,. push notifications, direct messaging, integrated video player that adjusts video quality based on bandwidth, easy-to-navigate platform), and functionality across mobile device operating systems (iOS, Android). The weekly psychoeducational videos, short symptom monitoring surveys, and social support community forum are housed seamlessly within the BEAM app. Other aspects of the program include individual check ins with trained peer coaches, group drop-in sessions that are facilitated by peer coaches where experts may be invited to discuss topics of interest (e.g., child psychologist, speech pathologist, paediatrician), and a connection to a Systems Navigator, whose role will be to support participants in accessing resources in their community. See Fig. 1 for an overview of all BEAM program components.

### Objectives {7}

The objective of the present intervention study is to conduct an effectiveness-implementation hybrid trial with co-primary aims of (1) determining BEAM’s effectiveness in improving parent mental health challenges (2) evaluating the implementation of BEAM in community agencies through metrics of feasibility, acceptability, and uptake. With respect to co-primary aim (1), we hypothesize that enrolled parents will evidence improvements in self-reported mental health (primary outcome) across four timepoints. Additionally, we hypothesize that enrolled parents will display reductions in parenting stress and harsh parenting behaviours, and report improvements in child mental health and developmental outcomes (secondary outcomes) across four timepoints. With respect to co-primary aim (2), we hypothesize that BEAM will meet quantitative and qualitative benchmarks of feasibility, acceptability, and program uptake that will motivate implementation of BEAM at greater scale.

We additionally aim to evaluate BEAM’s effectiveness in improving long-term health and psychosocial child and family outcomes relative to two population-based control cohorts. Using a natural experiment, we will construct ‘population-average’ and ‘risk-matched’ control cohorts from administrative data in the province of Manitoba, Canada. The ‘population-average’ cohort will consist of parents who match the basic demographics of BEAM participants and their children (e.g., age, number of children, etc.), while the ‘risk-matched’ cohort will consist of parents who match the basic demographics as well as the physical health, mental health, developmental, and service utilization characteristics of BEAM participants and their children (e.g., vaccination records, mental illness diagnoses, school literacy/numeracy benchmarks, hospitalization records). Administrative data will be drawn from childbirth (as early as January 2018) to as much as five years post-intervention (with an end date of June 2030). We hypothesize that participants who receive BEAM will evidence improvements in administrative measures of physical health, mental health, and developmental outcomes relative to the population-average and risk-matched cohorts.

The current study also includes the examination of baseline moderators (e.g., discrimination experiences, adverse child experiences) and exploratory variables (e.g., positive parenting, social support) which may be examined as exploratory outcomes, moderators, or mechanisms of change. Additionally, exploratory health economic outcomes pertaining to cost-utility will be examined (e.g., program delivery cost per quality-adjusted life-year [QALY]). With the goal of maximizing population health, a cost-utility analysis will compare BEAM’s delivery cost per QALY to that of extant programming [36, 37]. Other exploratory aims may be developed based on identified need and interest from partner community organizations, knowledge users, and collaborators.

### Trial design {8}

**Fig. 2:**
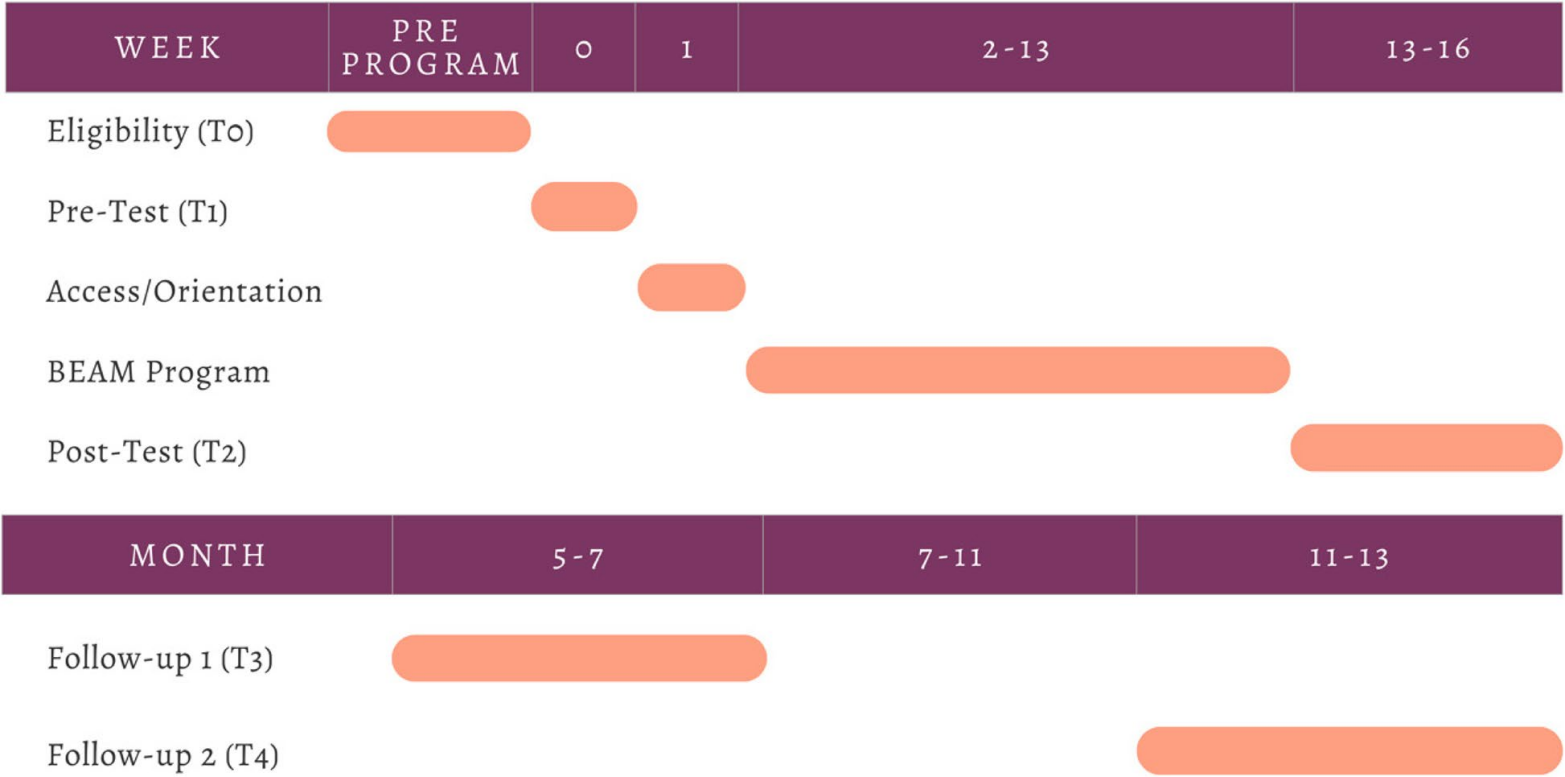
Participant Timeline

An effectiveness-implementation hybrid trial with repeated measures will be used to evaluate the efficacy of the 12-week app-based BEAM intervention for mental health and parenting outcomes for parents of children aged 24–71 months (at study enrolment, T0). In this research trial, parent is defined as anyone identifying as a primary caregiver to a young child (e.g., biological parent, foster parent, other relative). Timepoints of measures collected through the 12-week program are outlined in Table 1. Primary (parent mental health, child mental health, overall development) and secondary (child mental health, child development, parenting stress, harsh parenting behaviours) outcomes will be assessed immediately before start of the BEAM intervention (pre-test, T1), after the last week of BEAM (post-test, T2), at a 6-month follow-up (T3), and at a 12-month follow-up (T4); see Fig. 2 for participant timeline. We will also examine changes in physical health, mental health, development, and service utilization relative to parents in the community, as measured using de-identified administrative-linked data from provincial government health and social service use records in partnership with Manitoba Centre for Health Policy (MCHP). These administrative outcome measures will be examined from childbirth (as early as January 2018) up to five years after the trial (with an end date of June 2030). Other exploratory aims of this trial may include the evaluation of exploratory outcomes, sub-groups, moderators, and mechanisms of change. These exploratory analyses will only be examined in consultation with partner community organizations, knowledge users, and collaborators when there is identified need or interest.

**Table 1:** Effectiveness Outcomes

Construct	Metric	T0	T1	T2	T3	T4
Parent Mental Health	Patient Health Questionnaire 9-item	X	X	X	X	X
	Generalized Anxiety Disorders Questionnaire 7-item	X	X	X	X	X
	PROMIS Anger 5a	X	X	X	X	X
	PROMIS Sleep Disturbance 4a		X	X	X	X
	Alcohol Use Disorders Identification Test		X	X	X	X
	Cannabis Use Disorders Identification Test		X	X	X	X
Harsh Parenting	Parenting Stress Index – 4 th Edition – Short Form	X				X
	The Parenting Scale (PS)—Overreactive Discipline subscale		X	X	X	X
Child Mental Health	Child Behavior Checklist		X	X	X	X
Child Socioemotional Development	Ages and Stages Questionnaire: Socio-Emotional Second Edition		X	X	X	X
Child School Readiness	Ages & Stages Questionnaire-3		X	X	X	X
Positive Parenting	Parenting Self-Efficacy, 4-items from PSI-SF		X	X	X	X
Social Support	Social Support Effectiveness Questionnaire		X	X	X	X

This trial protocol reporting adheres to the Standard Protocol Items: Recommendations for Intervention Trials (SPIRIT) guidelines [38] and was registered with ClinicalTrials.gov. All procedures will be performed in accordance with the Research Ethics Board at the University of Manitoba, Fort Garry campus and the 1964 Helsinki Declaration and its later amendments. All participants will provide informed consent prior to enrolment in the study.

## Methods: participants, interventions and outcomes

### Study setting {9}

The intervention will be delivered through an app-based psychoeducation platform, hosted in Canada on secure Google Cloud Services in the Montréal Google Cloud Platform Region. Assessments (T0-T4) will be conducted online via the Research Electronic Data Capture (REDCap) tools for Personal Health Information Act (PHIA) compliance and data security [39–41].

### Eligibility criteria {10}

#### Inclusion and exclusion criteria

Eligibility includes: identifying as a parent with a child aged 24–71 months, English speaking, at least the age of 18 years, and residing in Manitoba, Canada. Parents must also self-report: moderate-to-severe symptoms of depression (≥ 10) on the Patient Health Questionnaire (PHQ-9) [42], and/or anxiety (≥ 10) on the General Anxiety Disorder (GAD-7) scale [43], and/or clinical levels of parenting stress (total score ≥ 110, parental distress subscale ≥ 38, parent–child dysfunctional interactions subscale ≥ 31, difficult child subscale ≥ 38) on the Parenting Stress Index – 4 th Edition – Short Form (PSI-4-SF)) [44], and/or clinical levels of anger (≥ 16) using the Patient-Reported Outcomes Measurement Information System (PROMIS-Anger) [45] at time of enrolment (T0). Parents with no self-reported symptom scale elevations but who are otherwise eligible will meet with a member of the BEAM team to determine eligibility. This eligibility decision will be based on clinical judgement, ensuring that those with need can participate. Eligible parents will be asked if they are available and willing to join the 12-week program and whether they can participate in regular individual check ins (15 to 60 min) via videoconference, phone, or direct messaging with a peer coach.

In terms of exclusion criteria, given that the BEAM program is not designed to support the needs of individuals at high risk for suicide and/or self-harm, parents who report self-harm that required medical attention within the past six months, a suicide attempt within the past year, or active thoughts of suicide with a plan will be ineligible without additional supports in place. These parents must be engaged in or on a waitlist for individual therapy with a psychiatrist, psychologist, social worker, or regulated mental health professional for the duration of their BEAM involvement. Parents at risk for suicide or self-harm who are not actively engaged in individual therapy will meet with a member of the BEAM team to be connected with or placed on a waitlist for these services before engaging in the BEAM program. Individuals who have participated in prior versions of the BEAM program will be excluded.

#### Screening and enrolment

After informed consent for the screener is obtained, prospective participants will be asked to complete an online eligibility screener to confirm that they are 18 years or older, speak English, have at least one child between 24 and 71 months, meet the threshold for depression, anxiety, parenting stress, and/or anger, respectively, and are willing to complete check ins with a peer coach (T0). Participants will be considered enrolled after they consent to participate in the study, complete the pre-test assessment, and participate in an orientation meeting with their peer coach and/or a member of the research team (T1). Individuals who do not meet the eligibility criteria will be excluded.

### Who will take informed consent? {26a}

Prospective participants who agree to be involved in the study will read an online informed consent form explaining the study procedures in full detail and will provide electronic written consent. Prospective participants will have the option to contact the research coordinators through the BEAM program email address should any questions regarding the informed consent form arise.

## Interventions

### Intervention description {11a}

The BEAM Program is built upon mHealth best practices and evidence-based program design principles, with the core objectives of improving parental mental health and fostering supportive parenting. The program's delivery will be facilitated through a mobile application, developed and overseen by MindSea. The BEAM program is a 12-week app-based mental health and parenting intervention, appropriate for the treatment of transdiagnostic emotion-based mental health symptoms (e.g., mood, anxiety, anger). In the 12-week self-guided BEAM program, participants will complete modules with a cohort of 40 to 80 parents. Each week includes: (a) 15–20 min of therapeutic video content, (b) exercises to practice key skills, (c) access to the online forum to cultivate social support through interaction with other parents and peer coaches, (d) individual check ins with peer coaches (i.e., trained and supervised staff with lived experience, employed by the University of Manitoba) via videoconference, phone, or direct messaging, lasting between 15 to 60 min depending on needs and preferences of participants, (e) a brief in-app survey for symptom monitoring to track progress across well-being and parenting metrics. Over the course of the program, participants will be invited to optional group drop-in sessions (e.g., tea and chat) facilitated by peer coaches on Zoom for Healthcare to connect and discuss program content and/or invite experts to discuss and answer questions for topics of interest identified by participants. Participants will also be connected with a Systems Navigator who can support them in accessing other resources (e.g., childcare programs, legal aid, housing) within their community. Peer coaches will be supervised at arm’s length by our clinical research team and will monitor the forums (i.e., encourage discussion, provide support) and lead all check ins with participants. The clinical research team will train peer coaches to flag signs of acute mental health risks, such as suicidal ideation, in which case the clinical team will assess the next steps for supporting the parent (e.g., crisis support resources). Additionally, peer coaches will be trained to deliver the BEAM program with fidelity by the research team (including recruitment, enrolment, participant tracking, coaching, and all aspects of delivery) using materials we have developed. The BEAM program aims to address the pressing need for accessible and effective mental health interventions by adopting a stepped care model. Stepped care is a comprehensive framework that offers a structured approach to providing mental health services in a tiered manner, ensuring that participants receive appropriate levels of support based on the severity of their needs. Within BEAM, at the heart of this model are peer coaches, who will have the most direct contact with participants. Peer coaches will monitor the online forum, facilitate individual check ins, and facilitate group drop ins. Peer coaches are supervised directly by a registered clinical psychologist, and will be trained to escalate concerns to our clinical team (i.e., clinical psychologists, senior students in clinical psychology) as needed.

Core components of the BEAM program are more thoroughly described below:

Each week, 15 to 20 min of therapeutic video content will be released on the BEAM mobile application. Videos will consist of psychoeducational parenting and mental health content. Parenting content was developed by Dr. Roos using emotion-focused parenting, parent management training, and attachment parenting theoretical orientations. Mental health content was developed by Dr. Roos and Dr. Tomfohr-Madsen drawing from transdiagnostic emotion-focused mental health and third wave Cognitive Behavioural Therapy principles such as Dialectical Behavioural Therapy, Acceptance and Commitment Therapy, and the Unified Protocol. Short weekly exercises will be released within the app alongside the therapeutic video content. Participants will be able to open the exercises and complete it using their device. The purpose of these exercises is to allow for the opportunity to practice key skills and consolidate program content.

Participants will complete a brief survey every week (i.e., modified symptom rating sliding scale, see Appendix 1; Appendix 2) within the BEAM app for symptom monitoring and feedback informed treatment. Each completed weekly survey will populate a graph in the app, displaying the trends of the participants’ symptom scores over time. This graph is visible to researchers, participants and their peer coach and can be discussed during check ins.

Each participant will have individual 15 to 60-min check-ins with a peer coach. These check-ins will occur at a frequency defined by the participant in consultation with the peer coach, and may occur anywhere from weekly to monthly. The first check-in of the program will be an orientation, where program goals are reviewed and participants can have their questions answered. Subsequent check ins will have coaches asking participants how they are feeling as they progress through the program materials and content. These check-ins are intended to support program progression and to answer any program-related questions. Participant attendance at these check ins will be tracked. Check-ins will take place through Zoom for Healthcare, phone, or direct messaging based on participant preference, needs, and availability. Participants will schedule their check-in using the scheduling software Calendly. Participants will be sent a link with the peer coach’s monthly availability and will be asked to input their first name and personal email to schedule an appointment. Calendly invitee data (i.e., first name and email) is hosted by Google and Amazon Web Services in the United States. Calendly data will not be linked to the BEAM app or BEAM data in any manner.

Participants will also have access to the app-based online forum to cultivate social support through interaction with other parents and peer coaches. The forum will be monitored by peer coaches who will also be active in the forum providing support and prompting discussion. Peer coaches will also facilitate optional drop-in groups via Zoom for Healthcare to provide an additional opportunity for participants to connect with peers and discuss program content.

All participants will be connected to a Systems Navigator who can support participants in accessing a wide variety of services. The Systems Navigator can connect participants to community resources such as housing supports, couples counselling, fun kids summer activities, culturally aligned resources and programming, and meet ups at community centres for to support ongoing relationship building. To ensure that the implementation of this Systems Navigator service aligns with the needs of our community partners, we hosted a Knowledge Exchange event with front-line service providers and community workers. At this event, we discussed the acceptability and feasibility of connecting their organizations and/or resource centres with a Systems Navigator. We will work to implement their feedback to ensure the Systems Navigator role appropriately supports parents and families. Additionally, we are building reciprocal relationships with community organizations where they will recommend parents to the BEAM program and our System Navigator will recommend parents back to their organizations for community services.

### Criteria for discontinuing or modifying allocated interventions {11b}

Should participants disclose suicidal behaviour, peer coaches will notify the clinical team. The clinical team will meet independent of the peer coaches to advise on whether or not continued participation in the BEAM program is in the best interest of the participant. In accordance with Best Practice Guidelines for Telepsychology Services, clinical research staff may also decide to terminate a participant’s involvement in the mHealth program if they deem it inappropriate for the participant to continue (e.g., if the participant cannot ensure confidentiality is maintained through access to a secure space and stable internet connection) [46]. Furthermore, participants may have discontinued access to the forum and may be denied access to the drop-in sessions if they engage in repeated violations of the terms of use. In these cases, participants would still have access to psychoeducation materials and would still be invited to do the assessments. The peer coaches may also provide a referral to another provider or clinic if deemed appropriate.

### Strategies to improve adherence to interventions {11c}

The proposed trial incorporates strategies such as reminders via in-app messaging, email, and telephone (SMS), which have also been shown to increase engagement [47]. Participants will be contacted through email by their peer coach if they have not attended check ins or engaged with the program material. Peer coaches will also be assigned to facilitate and promote participant engagement on the forum.

### Relevant concomitant care permitted or prohibited during the trial {11 d}

Participants will be permitted to receive concomitant care for mental health problems, such as psychotherapy, medication, and/or support groups. No concomitant care and interventions are prohibited. Concomitant care and interventions will be measured so they can be controlled for in analyses.

### Provisions for post-trial care {30}

If further treatment is deemed necessary, a referral to another provider or clinic will be made, and a list of community services will be provided.

### Outcomes {12}

#### Primary outcomes

This trial has co-primary aims evaluating: (1) BEAM’s effectiveness in improving parent mental health outcomes, and (2) the implementation of BEAM in the community through metrics of feasibility, acceptability, and uptake.

The co-primary effectiveness outcome is mean change in a composite score of parent mental health symptoms across all four timepoints: pre-intervention (T1), post-intervention (T2), 6-month follow-up (T3), and 12-month follow-up (T4). This composite score will be defined uniquely for each participant using a weighted average of their pre-intervention (T1) mental health profile (i.e., self-report symptoms of depression, anxiety, anger, and sleep problems). Pre-intervention symptoms above established clinical cut-offs will be mean-centred, standardized, and included in the participant’s composite mental health symptom score weighted by the symptom’s pre-intervention severity. In this way, the primary outcome will track mean change in each participant’s most clinically elevated pre-intervention symptoms. Depressive symptoms will be assessed using the PHQ-9 [42], a 9-item self-report measure of depression severity. Each item taps the frequency of depressive symptoms over the past week on a 4-point Likert scale (0 = *not at all* to 3 = *nearly every day*). Total scores range from 0 to 27, with higher scores indicating greater depressive symptom severity. A PHQ-9 cut-off score of ≥ 10 will identify participants with clinically elevated symptoms of depression. Anxiety symptoms will be assessed using the GAD-7 [43], a 7-item self-report measure of anxiety severity. Each item taps the frequency of anxiety symptoms over the past week on a 4-point Likert scale (0 = *not at all* to 3 = *nearly every day*). Total scores range from 0 to 21, with higher scores indicating greater anxiety symptom severity. A GAD-7 cut-off of ≥ 10 will identify participants with clinically elevated symptoms of anxiety. Anger will be assessed using the PROMIS Short Form, a 5-item self-report measure of anger frequency and severity [45]. Total scores range from 5 to 25, with higher scores indicating more anger. A PROMIS cut-off ≥ 16 will identify participants with clinically elevated anger symptoms. Sleep problems will be measured using the PROMIS Sleep Disturbance Scale, an 8-item self-report measure of sleep disturbances [48]. Total scores range from 8 to 40, with higher scores indicating greater severity of sleep disturbance. A PROMIS Sleep Disturbance Scale cut-off ≥ 30 will identify participants with clinically elevated sleep disturbances. All outcome measures are outlined in Table 1. We have obtained the necessary licenses to use all instruments mentioned in the protocol, ensuring compliance with their respective copyright and usage requirements.

The co-primary implementation metrics of feasibility, acceptability, and uptake when BEAM is implemented in the community will be assessed relative to previous trial benchmarks. Feasibility of the BEAM program will be assessed at post-intervention using the mHealth App Usability Questionnaire (MAUQ) [49] and other questionnaires developed for the BEAM program. Acceptability and uptake of the BEAM program will be assessed in three ways: (1) rates of recruitment, retention, (2) qualitative analysis of responses to post-intervention focus group questions that probe barriers and facilitators to program engagement, and (3) general program engagement measures from back-end app data. A list of implementation outcomes are outlined in Table 2.

**Table 2:** BEAM Implementation and Engagement Measures

Construct	Metric
***Back-end data tracking***	
Video Engagement	Total time spent watching videos
Forum Engagement	• Number of posts made on forum • Number of comments made on forum
Other	Exploratory measures pending feasibility in the app
***Coach/Research Team tracking***	
Weekly Survey Completion	Number of weekly surveys completed
Coach Engagement	• Number of 1:1 sessions attended • Number of times contacting coach and the type of contact
Drop-in Session Engagement	Number of drop-in sessions attended
Systems Navigator Engagement	Number of times systems navigator was engaged, and the types of support provided
***Self-Report Measures***	
App Usability	Self-report: mHealth App Usability Questionnaire
Engagement	Survey questions developed for BEAM
Satisfaction	Survey questions developed for BEAM
Therapeutic Alliance	Brief Revised Working Alliance Inventory
***Focus groups***	
Program Feasibility, Acceptability, Barriers, Facilitators	Open-ended questions developed for BEAM

#### Secondary outcomes

Secondary outcomes include mean change in parent mental health symptoms, harsh parenting behaviour, child mental health symptoms, child development, and child school readiness across all four timepoints: pre-intervention (T1), post-intervention (T2), 6-month follow-up (T3), and 12-month follow-up (T4). Change in parent mental health symptoms of depression, anxiety, anger, and sleep problems will be respectively measured by the PHQ-9, GAD-7, PROMIS Short Form, and PROMIS Sleep Disturbance scales, as previously defined [42, 43, 45, 48]. Mean change in substance use will also be assessed using the 10-item Alcohol Use Disorders Identification Test (AUDIT) [50] and 8-item Cannabis Use Disorders Identification Test – Revised (CUDIT-R) [51]. The AUDIT measures frequency of alcohol consumption, drinking behaviours, and alcohol-related psychological features. Total scores on the AUDIT range from 0 to 40, with higher scores indicating more severe alcohol use problems. The CUDIT-R measures frequency of cannabis consumption, problems, dependence, and cannabis-related psychological features. Total scores on the CUDIT-R range from 0 to 40, with higher scores indicating more severe cannabis use problems.

Other secondary measures include mean change in parenting stress and harsh parenting across all four timepoints: pre-intervention (T1), post-intervention (T2), 6-month follow-up (T3), and 12-month follow-up (T4). Mean change in parenting stress will be assessed using the 36-item PSI-4-SF [44], a self-report measure of parental stress due to individual parent and child factors as well as parent–child interactions. Total PSI-4-SF scores range from 36 to 180, with higher scores indicating higher levels of parenting stress. Mean change in harsh parenting disciplinary practices will be assessed using the Parenting Scale [52], a 30-item self-report measure that assesses parenting behavior and dysfunctional discipline in parents with young children. Total scores range from 30 to 210, with higher scores indicating higher levels of ineffective discipline practices.

Mean change in child mental health, development, and school readiness will similarly be measured across all four timepoints. Child mental health symptoms will be assessed at each timepoint using the CBCL [53], a parent-report questionnaire that is used to detect behavioural and emotional problems in children. The CBCL demonstrates adequate internal reliability (α’s ≥ 0.78) and has precedent as a measure of change in intervention research [46, 54–56]. The CBCL includes 99 items in which parents are asked to rate their child on how true each item is from 0 (*not true*) to 2 (*very true or often true of the child*). Possible scores range from 0 to 198, where higher scores indicate more mental health symptoms. Items are scored on the following syndrome scales: emotionally reactive, anxious/depressed, somatic complaints, withdrawn, attention problems, aggressive behaviour, and sleep problems. The CBCL total score and syndrome scores will be examined. Change in child socioemotional development will be assessed using the Ages and Stages Questionnaires: Socioemotional, Second Edition (ASQ:SE-2), a screening tool that identifies socioemotional challenges across a range of developmental domains [57]. Different versions of the ASQ:SE-2 exist depending on the age of the child. For this trial, five versions of the ASQ:SE-2 cover the age of all eligible children. Depending on version, the ASQ:SE-2 has approximately 30 items which sum to a total score. Higher scores represent more severe socioemotional challenges. Change in child school readiness will be measured by the Ages and Stages Questionnaire, Third Edition (ASQ-3), a parent-report screening tool that taps child developmental progress across domains of communication, motor, problem solving, and personal-social [57]. Nine versions of the ASQ-3 cover the age of all eligible children. The ASQ-3 contains 30-items which sum to a total score. Higher scores represent a greater number of met developmental milestones, indicative of school readiness.

#### Exploratory outcomes

Exploratory outcomes include changes in positive parenting behaviour and social support [58]. Change in positive parenting will be measured by four items of the PSI-4-SF that tap parenting self-efficacy [44, 59, 60]. Change in social support will be measured using the SSEQ [61]. Total scores range from 0 to 80, with higher scores representing greater social support effectiveness [57].

Exploratory mediators and moderators of change include discrimination, adverse childhood experiences, concomitant service utilization, food insecurity, housing instability, therapeutic alliance, and participant sociodemographic and socioeconomic characteristics (Table 3). Discrimination will be measured using the Everyday Discrimination Scale (EDS), a 5-item self-report scale measuring subjective experiences of discrimination [62]. Adverse childhood experiences will be assessed with the Adverse Childhood Experiences (ACEs) questionnaire. [63]. Total scores range from 0 to 10, with higher scores indicating more adverse experiences in early childhood. Concomitant service utilization will be measured by a self-report questionnaire developed for the BEAM program that is meant to supplement the collected administrative data. The service utilization questionnaire asks about the total number of contacts and total contact hours with a variety of acute (e.g., hospitalization), primary (e.g., family doctor visit), and community-based services (e.g., child care centres, family resource centres). Food insecurity will be assessed by the Household Food Security Survey Module (HFSSM) from the Canadian Community Health Survey [64]. The HFSSM taps household food insecurity over the past 12 months. Total scores range from 0 to 16, with higher scores indicating greater food insecurity. Such use of questionnaires from the Canadian federal government will enable comparison of BEAM participant food security and housing stability with Canadian population averages. Housing instability will be measured by the Housing Instability Scale (HIS) [65]. The HIS taps housing instability over the past 6 months. Total scores range from 0 to 7, with higher scores indicating greater housing instability [66]. Therapeutic alliance between the peer coaches and participants will also be assessed using the Brief Revised Working Alliance Inventory (BR-WAI), a 16-item measure assessing bonds, tasks, and goals within the therapeutic relationship [67]. Finally, participant sociodemographic and socioeconomic information will be assessed by a questionnaire developed for the BEAM program. This questionnaire asks about parent, child, and family demographics, household income, parent marital status, educational attainment, employment status, and childcare.

**Table 3:** Sociodemographic and Socioeconomic Variables

Construct	Metric	T0	T1	T2	T3	T4
***Self-Report Measures***						
Sociodemographic information (constructs include age, income, marital status, education, …)	Sociodemographic Questionnaire (developed for BEAM)		X	X	X	X
Service Utilization (constructs include contact hours with acute, primary, community-based care services)	Service Utilization Questionnaire (developed for BEAM)		X	X	X	X
Food Security	Household Food Security Survey Module (HFSSM; 2024 Canadian Community Health Survey)		X	X	X	X
Housing Stability	Housing Instability Scale (HIS)		X	X	X	X
Discrimination	Everyday Discrimination Scale (EDS)		X	X	X	X
Childhood Experiences	Adverse Childhood Experiences		X			

### Administrative measures

This trial of BEAM will also examine changes in family health and education, as measured using de-identified administrative-linked data from provincial government health and social service records in partnership with the Manitoba Centre for Health Policy (MCHP). This means that participant self-report data collected as part of the BEAM program will be linked to their provincial government health and social services records from the Manitoba Population Research Data Repository. Firstly, this linkage enables the longitudinal analysis of a broader scope of participant family physical health, mental health, and education outcomes from childbirth to up to five years post-intervention (June 2030). Secondly, this linkage enables the longitudinal comparison of BEAM participants to two population-based cohorts (‘population-average,’ ‘risk-matched’ control cohorts) of families who did not participate in BEAM, allowing examination of how BEAM may lead to lasting family improvements in psychological, social, and physical health. Participants are not required to consent to administrative data linkage to participate in BEAM. Those who do not consent will be excluded from the BEAM population-based cohort, but there is no way to ensure that they will be excluded from a control cohort. This is because those who opt out may not provide sufficient demographic information (e.g., Personal Health Identification Number [PHIN], age, postal code) to ensure control cohort exclusion. Preliminary data shows more than 80% of participants opting in. Further, every possible step will be taken to remove any BEAM participants from the control cohorts (e.g., by cross-referencing participant full names). Our analysis will be securely conducted by trained analysts from the Manitoba Centre for Health Policy (MCHP), the custodians of the Manitoba Population Research Data Repository.

Administrative data will include measures of family health and wellbeing (i.e., socioeconomic status, perinatal health, mental health, and substance use) assessed through the Families First Home Screening Form. Parent medical care including, hospitalizations, emergency visits, prescription medications, physical illness (e.g., atopic dermatitis), mental illness (i.e., mood, anxiety, substance use, psychotic disorders), and cause of death will be assessed using Hospital Abstracts Data, Medical Services Data, the Mental Health Management Information System (MHMIS), the Drug Program Information Network (DPIN), and Vital Statistics Mortality Registry Data. Child medical care including traumatic injury, hospitalizations, emergency visits, physical illness (e.g., lower respiratory tract infection), mental and neurodevelopmental disorders (e.g., mood, anxiety, trauma-related disorders, ADHD), prescription medications, and vaccinations will be assessed using the Mental Health Management Information System (MHMIS), Medical Services Data, and Hospital Abstracts Data, and the Manitoba Immunization Registry. Measures related to child development including age-appropriate grade, literacy, numeracy, special needs funding status, academic accommodations, health and psychosocial development, child care centre use, family income assistance, and family employment will be measured through enrolment, Marks and Assessment (STS/ICAB), Early Developmental Instrument (EDI), Child Day Care Program, and Manitoba Education Special Needs. Finally, Child Family Services (CFS) and parent justice involvement including days in CFS care, charges, and charge categories may be examined using the Child and Family Services Information System (CFSIS) and Justice Prosecution Information and Scheduling Management (PRISM), pending indication of interest and approval from the First Nations Health and Social Secretariat of Manitoba’s Health Information Research Governance Committee. Administrative data will also include basic demographic information such as date of birth, biological sex, postal code of residence, and family registration number. These data will be drawn from the Manitoba Health Insurance Registry [49, 67].

### Participant timeline {13}

SPIRIT schedule of enrolment, intervention, and assessments.

Participant timeline is outlined in Fig. 2.

Eligibility (T0): Parents of young children may complete the eligibility screener to determine whether they are eligible for participation in the BEAM program.

Week 0 (T1): Eligible participants who consent to participate in the program will receive notice of enrolment. Participants will be fully enrolled in the program after they participate in an orientation meeting with their peer coach and/or a member of the research team and complete the pre-test assessments, including primary, secondary, and exploratory outcome measures.

Week 1: Participants will receive an email regarding BEAM app access login and account information. Program information will be sent electronically to participants from the study team.

Weeks 2–13: Participants will watch weekly videos and will complete symptom tracking questions for self-monitoring purposes. Participants will begin engaging in the forum and will also participate in check ins with their peer coach, continue engaging in the forum, and connect with the Systems Navigator as needed.

Weeks 13–16 (post, T2): Participants will complete a full post-test assessment of primary, secondary, and exploratory outcome measures.

Month 5–7 (follow-up, T3): Participants will complete a follow-up assessment of primary and secondary outcome measures.

Month 11–13 (follow-up, T4): Participants will complete a follow-up assessment of primary and secondary outcome measures.

### Sample Size

In total, we aim for *N* = 400 participants to participate in the BEAM program over two years. However, we may modify or extend eligibility based on participant attrition rates and budgetary capacity. Sample size was determined based on data from our telehealth pilot in preschoolers and related effectiveness trials comparing evidence-based practices to services as usual in treatment seeking families [68]. We assume that participation in BEAM will evidence moderate reductions in parent mental health, based on prior evidence from previous BEAM trials. For this effectiveness trial, we anticipate the effect size may be approximately 25% smaller than in trials delivered directly by our practitioner team. The sample size calculation was conducted to ensure adequate power to detect this reduced effect size. Specifically, we conducted a power analysis assuming a type I error rate (α) of 0.05 and a power (1-β) of 0.80, which corresponds to a 80% probability of detecting a statistically significant effect if it exists. The calculation was based on a linear mixed-effects model, which accounts for repeated measures over time. To account for potential loss to follow-up, we conservatively assumed an attrition rate of 30%.

With these parameters, we determined that a sample size of N = 400 participants would provide sufficient power to detect the expected effect size while maintaining robustness against attrition and variability in outcomes. This calculation ensures the trial is appropriately powered for the primary analysis of intervention effectiveness.

### Recruitment

Recruitment will involve a multi-faceted approach, encompassing four primary recruitment streams. First, we will collaborate closely with Manitoba’s Shared Health Crisis Response Services, including programs offering crisis assessment and post-crisis intervention to individuals across the province, to engage front-line staff in the recruitment process. Front line staff will receive training to enhance their familiarity with BEAM as a resource. The training will include comprehensive insights into BEAM program specifics, eligibility criteria, as well as introduce staff to our BEAM Systems Navigator and the stepped care model. Secondly, we will target recruitment efforts at primary care offices and provincial health services (e.g,. Shared Health Mental Health Services), in consultation with the Manitoba Pediatric Society and Doctors Manitoba. Recruitment through this stream will utilize light touch strategies such as poster and pamphlet drop-offs to sites. The third recruitment avenue involves collaborating with various community organizations, child care centres, and family resource centres (e.g., Family Dynamics, United Way Winnipeg, Acorn Family Place). Through these partnerships, we aim to expand the reach of our recruitment efforts and ensure that families from diverse backgrounds are included in the trial. Finally, our online recruitment efforts will include sharing posters and short video advertisements on social media platforms (e.g., Facebook, Instagram). Through these various approaches, we aim to facilitate comprehensive recruitment and maximize the representativeness of the trial.

Participant screening and recruitment will be done on a continuous, rolling basis over two years, from December 2023 to September 2025.

### Assignment of interventions: allocation

Not applicable, there will be no assignment to different interventions.

### Assignment of interventions: blinding

Not applicable.

## Data collection and management

### Plans for assessment and collection of outcomes {18a}

Data will be collected via self-report measures, the use of back-end app engagement data, forum content, peer coach and system navigator meeting attendance, and administrative data. Primary outcomes, secondary outcomes, and exploratory outcomes are outlined in Table 1. There will additionally be measures of participant *sociodemographic information*, *service utilization*, *food insecurity*, and *housing instability*. These constructs will be assessed using questionnaires designed for BEAM and a questionnaire from the Canadian Community Health Survey to enable comparison of BEAM participant experiences with food insecurity to those of the Canadian population average. Peer coach check-in attendance, system navigator meeting attendance, and app analytics will be used to track program engagement and adherence. The content of posts and comments within the forum will be used for program improvement purposes. Administrative data records will be drawn from the Manitoba Population Research Data Repository, maintained by the Manitoba Centre for Health Policy (MCHP). All analyses will be completed by MCHP analysts and no person-level administrative data will be accessible to our research team.

### Plans to promote participant retention and complete follow-up {18b}

Study-related reminders will be sent to participants when self-report questionnaires are being collected (T1-T4). Eligible participants will receive up to a total of $200 Canadian (CAD) in compensation for participating for the entire duration of the study, see Table 4. Participants will receive an honorarium of $10 CAD for completing the pre-assessment questionnaires, $30 CAD for completing the post-intervention assessment, $40 CAD for completing each follow-up assessment, and $10 CAD at each timepoint to complete a school readiness questionnaire. Participants will receive an additional $10 at post and follow-up timepoints if they complete the questionnaires within 2 weeks. Additionally, participants will receive $10 CAD if they complete half of all weekly surveys (6 or more of the 12 surveys). Furthermore, the clinical and research team will have weekly meetings to monitor the adherence and retention of participants. For instance, participant engagement will be discussed and additional email check-ins with participants will be performed when advised by the registered psychologists on the team.

**Table 4:** BEAM Program Participant Compensation

	Pre-program (T1)	Post-program (T2)	6 mo. follow-up (T3)	12-mo follow-up (T4)	Total
45-min questionnaire	$10	$30	$40	$40	$120
15-min school readiness questionnaire	$10	$10	$10	$10	$40
Complete questionnaires within 2 weeks		$10	$10	$10	$30
Complete at least 6 of 12 weekly in-app surveys	$10				$10
					**$200**

### Data management {19}

REDCap is managed by The George and Fay Yee Centre for Healthcare Innovation, which is a hired consultant on the proposed project and will support secure data collection and management. Data will be stored on a secure server in accordance with the University of Calgary's Data Retention Policy and the University of Manitoba's PHIA policies. Once data is downloaded, all identifying information will be removed, which aligns with the University of Manitoba's REDCap policies. All patient health information will be kept for up to 5 years. Birth dates will be converted into age in months, and birthdate information will then be destroyed. Measures to promote the quality of data entry will include range checks for all data values entered and having three research assistants review and ensure correct data entry in REDCap.

### Confidentiality {27}

The BEAM program will occur on a secure online platform and participant confidentiality will be protected throughout all phases of the study in accordance with University of Manitoba ethics guidelines. All questionnaire data will be stored securely on REDCap or on password-protected University of Manitoba secure data servers. Precautions will be taken to manage any risks to confidentiality. These precautions include a Risk Management Protocol, Data Management Protocol, and participant agreed upon Terms of Use. During the group drop-ins, participant anonymity cannot be guaranteed. These sessions will be hosted on a secure Zoom for Healthcare account and will be password-protected. The peer coaches will outline the limitations of confidentiality and anonymity at the beginning of each drop-in session. Drop-in sessions will not be recorded or included in any assessment; however, attendance will be tracked. Participants will become familiar with the limitations to confidentiality and of the measures that will be taken to protect the confidentiality during the informed consent process before beginning in the study.

### Plans for collection, laboratory evaluation, and storage of biological specimens for genetic or molecular analysis in this trial/future use {33}

Not applicable, no biological specimens will be collected.

## Statistical methods

### Statistical methods for primary and secondary outcomes {20a}

Analysis of the primary outcome (parent mental health symptom composite) will examine change across all four timepoints: pre-intervention (T1), post-intervention (T2), 6-month follow-up (T3), and 12-month follow-up (T4). A two-level linear mixed effects model with random slopes will evaluate the hypothesis that parent mental health symptoms will evidence significant reductions from T1 to T2, T1 to T3, and T1 to T4. This model will account for sociodemographic and socioeconomic factors as well as other theoretically- and empirically-motivated covariates [69]. Baseline mental health symptom severity (T0) will be examined as a baseline moderator. Other theoretically- and empirically motivated exploratory baseline moderators of change may also be examined. The same two-level linear mixed effects modelling approach will evaluate change in secondary outcomes (i.e., individual parent mental health symptoms, parenting behaviours, child mental health and development). All data will be included following an intention-to-treat approach, where missing data will be handled using full information maximum likelihood estimation. Sensitivity analyses will be used to examine complete-case methods should the assumption of missing at random be met. Analyses will be led by the research team, in consultation with the Centre for Healthcare Innovation, using MPlus v8.0. The research team will also investigate if there is baseline (T0) moderation by symptom severity to examine whether the BEAM intervention is more effective for participants who have higher symptom levels before receiving the intervention (e.g., depression, anxiety, anger, sleep disturbances).

To measure the impact of BEAM via implementation metrics and cost-effectiveness we will use benchmark uptake and attrition markers, to determine the extent to which the BEAM program succeeds on key implementation metrics including acceptability, feasibility, fidelity, cost, and sustainability [70]. Mixed-methods data from agency perspective, app usage, and user experience will inform continuous improvements using implementation metrics we have used in the past including app, group and coach engagement as well as user satisfaction. Quantitative data related to app usage and UX data will be linked to outcome variables to elucidate what components of program engagement are associated with symptom remission.

Analysis of administrative-linked outcomes will enable comparisons between BEAM families and those families in ‘population-average’ and ‘risk-matched’ control cohorts drawn from MCHP’s provincial administrative database. While the ‘population-average’ control cohort will be comprised of parents who match the basic demographics of BEAM participants and their children (e.g., age, number of children), the ‘risk-matched’ control cohort will consist of parents of young children who match the demographics, physical health, mental health, child developmental, and service utilization characteristics of BEAM participants and their children. This will be accomplished in consultation with MCHP by matching Manitoban families in the administrative database to BEAM families on these risk factors. These matching criteria will be developed using MCHP’s high-dimensional propensity scores matching algorithm (hdPSs) which, across multiple algorithmic iterations, identifies risk factors that are correlated with the features of the participants who have enrolled in the BEAM program [71]. This data-driven propensity score development strategy is commonly used and will allow our team to match BEAM participant risk to non-BEAM participants in the community. Comparing trajectories in health and social outcomes between BEAM participants, ‘risk-matched’ controls, and ‘population-average’ controls will generate knowledge about the extent to which children experience stable, cumulative, or “sleeper” (i.e., not observable until after developmental time periods) benefits over time. If we find children experience diminishing effects such that program benefits are only present in the first year, this may indicate that further intervention is required to sustain program achievements. All administrative-linked analyses will be conducted by MCHP statisticians.

Exploratory qualitative data will be analyzed thematically [38]. First, two coders will analyze the data concurrently, but independently read the transcripts for surface descriptive content, and organize like-with-like ideas. Second, transcripts will be re-read for latent meaning to better understand participants'meaning, including looking for contradictory or confirmatory statements. Each coder will independently generate and systematically apply themes across all transcripts. Semantic validity checks will ensure different words and phrases within a category have similar meanings [38]. To further enhance rigor, constructs will be deemed saturated (akin to statistical significance in quantitative work) when no new or additional constructs are identified, consensus is reached on all overarching themes, and no alternative explanations were found with subsequent interviews [38].

Exploratory health economic analyses will examine BEAM’s delivery cost per QALY and will compare cost per QALY to that of extant mental health programming [36, 37]. This analysis will be conducted in consultation with health economist and trial steering committee member (F Clement).

### Interim analyses {21b}

No interim analyses are planned.

### Methods for additional analyses (e.g., subgroup analyses) {20b}

Subgroup analyses may be explored based on emergent research questions of interest.

## Methods in analysis to handle protocol non-adherence and any statistical methods to handle missing data {20c}

Analyses will be conducted on an intent-to-treat (ITT) basis [72]. Regardless of participant attrition or withdrawal, data for every participant to the point of attrition will be included in ITT analyses. Missing data will be handled using maximum likelihood [73], which estimates values based on all available data and will produce unbiased model parameters given that the data are independently and identically distributed [74]. Sensitivity analyses will be used to examine complete-case methods should the assumption of missing at random be met.

### Plans to give access to the full protocol, participant-level data, and statistical code {31c}

The full trial protocol will be made publicly available through ClinicalTrials.gov. Dr. Leslie Roos will remain the custodian of the data, as the primary investigator. All primary data will be analyzed independently by the primary investigator’s team, and will not be released to any third party (including the funder) before the trial is completed. The exception to this are the administrative data analyses. These analyses will be conducted by MCHP, a department of the University of Manitoba, who will have access to the minimum amount of participant data that will allow analysis and participant data linkage to their administrative data. In accordance with the International Committee of Medical Journal Editor's guidelines, the authors will share de-identified patient self-report data that underlies results presented in reports no later than 6 months after publication. Participant-level administrative data will not be made available. The primary investigator will ensure all de-identified self-report data is available upon request.

## Oversight and monitoring

### Composition of the coordinating centre and trial steering committee {5 d}

Dr. Roos, as the primary investigator, will be responsible for the overall management of the project. Dr. Roos has expertise in maternal-child health interventions and will lead platform development and parenting content for participants. The project’s steering committee (SC) will consist of the co-leads (L Roos, M Braun), the EDI champion (L Tomfohr-Madsen), and the iKM lead (M Archibald) along with leaders in BEAM program development (A MacKinnon), applied population health and social determinants of health and health inequities (N Nickel) and health economics (F Clement). From its inception, BEAM has been co-designed with our patient-advisory board (PAB) of parents holding lived experience with mental illness and parenting stress. Steering committee members will be advised by the BEAM parent advisory board (led by A Pharazyn) and a research advisory board comprising experts in the areas of clinical trials and regulatory strategy (L Kelly, T Klassen), child health and developmental outcomes (G Giesbrecht, C Lebel, R Giuliano), child maltreatment (T Afifi), rural mental health (A Musquash), qualitative methods (K Reynolds), clinical health psychology child and adolescent services (J Unger), health services integration (L Katz) and implementation science (P Fisher). The steering committee will meet quarterly (in person or online) with updates sent monthly or as needed from the respective research sections. SC meeting agendas will include trial progress, preliminary outcome reporting, pitfall mitigation, and knowledge interpretation. Video development will be completed alongside WorkerBee.TV, industry developers specializing in strategic video development. The BEAM mobile application was redesigned and revised by expert app developers at Mindsea specializing in digital health program development. The research team will meet with each respective industry developer weekly.

The research team’s affiliation with the Children’s Hospital Research Institute of Manitoba (CHRIM) provides a platform for engagement with the institute’s Indigenous advisory council along with Elder Mary Wilson, a spiritual leader and Knowledge Keeper in the community. To include and give privilege to Indigenous voices across all stages of this work, we are committed to the respectful development of these relationships as well as many others through existing connections with community organizations, knowledge users, and collaborators (e.g., Family Dynamics, Huddle Manitoba, Strengthening Families Maternal Child Health, United Way Winnipeg, Acorn Family Place, Ma Mawi Chi Itata Centre). Applicants Mushquash, Klassen, Fisher, Katz, Archibald, Nickel, and Roos hold experience in partnerships with community leaders and agencies serving diverse populations.

We have begun to assemble a Community Advisory Board through existing and emerging relationships with individuals who have lived experience and knowledge of mental health care services, research development, data sovereignty, and traditional knowledge. This board (Council) will meet regularly, serving as advisors in all stages of research from design to dissemination. The Council will support the research team to promote equitable and ethical care of participants.

### Composition of the data monitoring committee, its role, and reporting structure {21a}

Study investigators will monitor the trial. There will be weekly meetings with the research and clinical teams and the primary investigator (L Roos) to discuss and review each phase of the trial (e.g., recruitment, data collection). There will also be weekly meetings with the peer coaches (i.e., those who are interacting with research participants) and the clinical team to discuss and resolve all potential questions and concerns of participants. Given the minimal risks of the program, short duration (12 weeks of intervention), and relatively small sample size, the current trial does not require a formal data monitoring committee.

### Adverse event reporting and harms {22}

No adverse experiences occurred in our work to date and we have a risk-management protocol in place for mental health crises or child maltreatment risk. Our ethics-board approved protocol includes team training in mandatory reporting requirements and emergency protocols as well as guidance on when to consult.

Additionally, no known risks to parents are associated with digital psychoeducational interventions. Should participants report significant distress or request additional support, peer coaches will follow up with these participants and consult with clinical supervisors using a risk-management protocol. The primary investigators will check in with peer coaches regularly to ensure that the study participants' questions and concerns are responded to appropriately. No adverse events were reported in our pilot or RCT study.

We will follow the University of Manitoba's standard procedures for reporting adverse events and protocol violations/deviations to the Research Ethics Office at the Fort Garry Campus and privacy breaches to the Access and Privacy Office.

### Frequency and plans for auditing trial conduct {23}

The Research Ethics Board may request an audit. However, the study team has no plans for independent auditing of trial conduct.

### Plans for communicating important protocol amendments to relevant parties (e.g., trial participants, ethical committees) {25}

Any amendments to the protocol will be submitted to the Ethics Board at the University of Manitoba, who will review and approve the amendments. If the amendment requires that revised information be communicated with enrolled research participants, this will be done through a consent addendum, which will be provided to participants through email. Any amendments to our protocol approved by the Ethics Board will be forwarded to Manitoba Shared Health, our recruitment partner, for their review. Additionally, any protocol amendments pertaining to administrative data linkage or collection of personal health information relevant to data linkage will be reviewed by the provincial body in charge of administrative data access (the Provincial Health Research Privacy Committee [PHRPC]).

### Dissemination plans {31a}

The results of our research will be shared with both academic and non-academic audiences within a year of completing the trial, regardless of the impact size or direction. We will prepare manuscripts for academic publication detailing the effects of the BEAM implementation trial on both primary and secondary outcomes. These papers will also be posted to preprint servers, ensuring open access to a wider audience. To reach diverse groups, our team, including principal investigators and trainees, will present these findings at both national and international conferences, such as the Canadian National Perinatal Research meeting and the Society for Research in Child Development. Additionally, we will disseminate information using infographics and bimonthly reports through collaborations with community agencies and established government contacts. Lay summaries will be available on our lab and study websites, as well as on social media platforms. Finally, we will host a knowledge sharing gathering for interested community collaborators and knowledge users. A meal will be provided to all attendees along with multi-media presentations of findings over a half-day event. Storytelling and discussion will be facilitated to promote shared learning and decolonial methods of dissemination.

Our principal knowledge user is Family Dynamics, a community agency that provides services to thousands of families in Manitoba. Family Dynamics has been the primary agency partner throughout the development of the BEAM program. Manitoba is an ideal province to test this model due to high rates of child mental health problems and low school readiness. Families hold varied experiences across rural/urban settings, and newcomer, Indigenous and Francophone identities are common. Our integrated knowledge mobilization (iKM) plan employs a “living lab,” a collaborative online platform designed to transform healthcare initiatives [75–77]. The BEAM Living Lab will facilitate long-term partnerships with diverse stakeholders through an easy-to-use online interface designed for knowledge sharing, priority setting, data interpretation, and dissemination activities.

## Discussion

Exposure to parental MI is associated with socio-emotional impairments and mental health problems in children. Since the COVID-19 pandemic, parental MI has increased threefold. Despite this rapid increase, many parents and caregivers do not access evidence-based parenting and mental health treatments [2, 3, 11]. Scalable and accessible solutions to promote mental health and support parenting is critical in improving developmental outcomes in at-risk children. The BEAM program is an evidence-based app program that shows promising results in reducing parental MI and harsh parenting practices. This trial will build on previous RCTs of the program with a focus on determining BEAM’s effectiveness in improving the mental health of parents with mental health challenges and who have a young child with mental illness and evaluating the implementation of BEAM in community agencies. Investing in parental mental health early and scaling interventions to be widely accessible through community organizations is expected to yield high economic benefits by preventing the long-term consequences of parental MI on child development. Findings from this RCT will improve our understanding of the effectiveness of BEAM and allow for testing of BEAM’s readiness for nationwide scaling.

### Trial Status

Recruitment began in early January 2024 and will be ongoing until expected finish in September 2025. The date of first enrolment was March 7, 2024; cohort 1 of the intervention was launched March 11, 2024. Estimated completion in January 2027 for the collection of all primary measures.

## Figures and Tables

**Figure:**
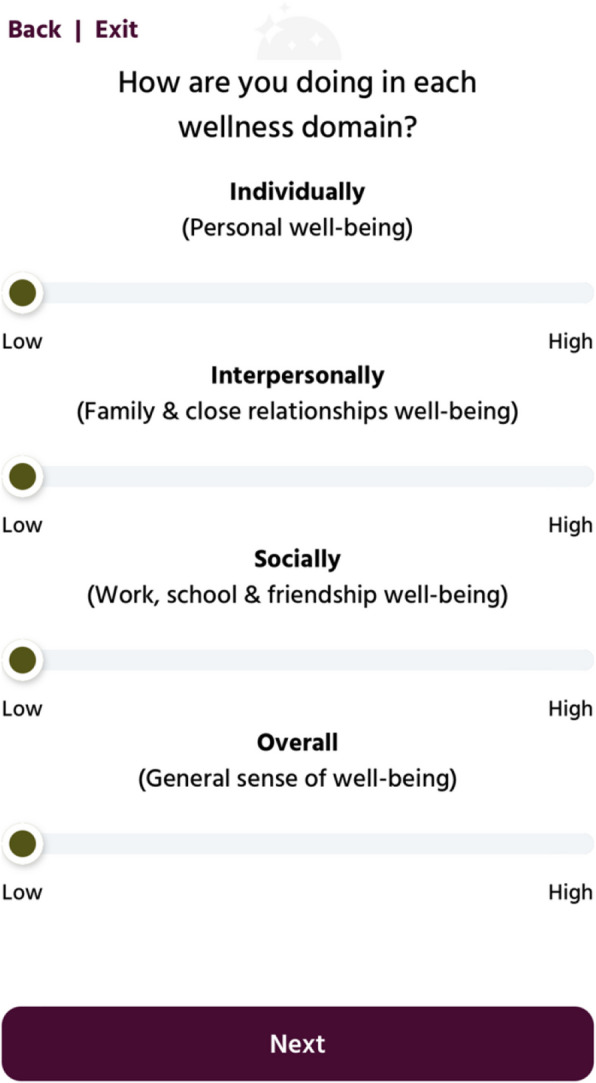
Well-being Rating Scale – Modified Symptoms Rating Sliding Scale

**Figure:**
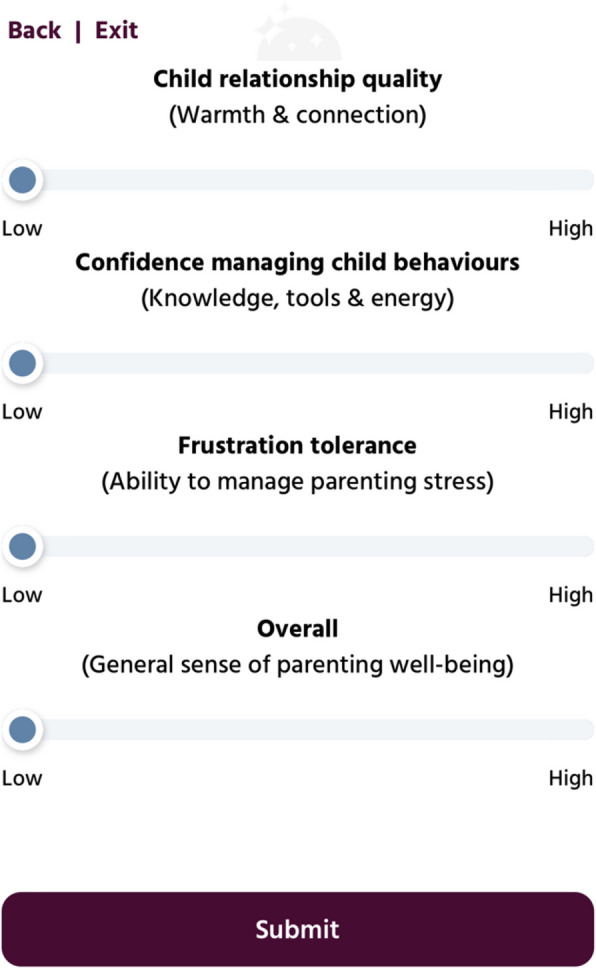
Parenting Rating Scale – Modified Symptoms Rating Sliding Scale

## Data Availability

The full trial protocol will be made publicly available through ClinicalTrials.gov. In accordance with the International Committee of Medical Journal Editor’s guidelines, the authors will share de-identified participant self-report data that underlies results presented in reports no later than 6 months after publication. All de-identified individual self-report data underlying results will be made available upon reasonable request. Requests for data should be directed to the trial sponsor (LER) at leslie.roos@umanitoba.ca. All requests should detail the reason for the request and describe how the data will be used.

## Abbreviations

ACEs: Adverse Childhood Experiences
ASQ:SE-2: Ages and Stages Questionnaires: Socioemotional, Second Edition
ASQ-3: Ages and Stages Questionnaires, Third Edition
AUDIT: Alcohol Use Disorders Identification Test
BEAM: Building Emotional Awareness and Mental Health (BEAM)
BR-WAI: Brief Revised Working Alliance Inventory
CBCL: Child Behavior Checklist
CFS: Child and Family Services
CHRIM: Children’s Hospital Research Institute of Manitoba
CUDIT-R: Cannabis Use Disorders Identification Test-Revised
DPIN: Drug Program Information Network
EDI: Early Developmental Instrument
EDS: Everyday Discrimination Scale
GAD-7: General Anxiety Disorder-7
HDPS: High-Dimensional Propensity Score
HFSSM: Household Food Security Survey Module
iKM: Knowledge Mobilization
ITT: Intent-to-treat
MHMIS: Mental Health Management Information System
MCHP: Manitoba Centre for Health Policy
mHealth: Mobile health
MI: Mental illness
PHRPC: Provincial Health Research Privacy Committee
PHQ-9: Patient Health Questionnaire-9
PSI-4-SF: Parenting Stress Index-4th Edition-Short Form
PRISM: Prosecution Information and Scheduling Management
PROMIS: Patient-Reported Outcomes Measurement Information System
QALY: Quality-Adjusted Life Year
RCT: Randomized Control Trial
REDCap: Research Electronic Data Capture
SAU: Services-as-usual
SC: Steering Committee
SSEQ: Social Support Effectiveness Questionnaire
